# Effects of resistance training on quality of life, fatigue, physical function, and muscular strength during chemotherapy treatment: a systematic review and meta-analysis

**DOI:** 10.1007/s00520-024-08766-y

**Published:** 2024-08-17

**Authors:** James W. Metcalfe, Samuel T. Orange, Leigh A. Madden, Phil Marshall, Rebecca V. Vince

**Affiliations:** 1 School of Sport, Exercise & Rehabilitation Sciences, Faculty of Health Sciences, University of Hull, Hull, UK; 2 School of Biomedical, Nutritional and Sport Sciences, Faculty of Medical Sciences, Newcastle University, Newcastle Upon Tyne, UK; 3 Newcastle University Centre for Cancer, Newcastle University, Newcastle Upon Tyne, UK; 4 Centre for Biomedicine, Hull York Medical School, University of Hull, Hull, UK

**Keywords:** Chemotherapy, Resistance Training, Quality of Life, Fatigue, Physical Function, Muscular Strength

## Abstract

**Purpose:**

To systematically review and meta-analyse the efficacy of resistance training on quality of life (QOL), fatigue, physical function, and muscular strength in people diagnosed with cancer undergoing chemotherapy.

**Methods:**

Electronic databases PubMed, Cochrane Central, CINAHL, SCOPUS and Web of Science were systematically searched for randomised controlled trials (RCTs) that compared the effects of resistance training to control on QOL, fatigue, physical function, and lower-body and upper-body muscular strength in adults undergoing chemotherapy. Standardised mean differences (SMDs) were pooled using a random effects model. Risk of bias was assess using the risk of bias tool for randomised trials (RoB 2).

**Results:**

Seven RCTs encompassing 561 participants were included. The pooled results of seven RCTs showed that resistance training during chemotherapy significantly improved lower-body strength (n = 555, SMD 0.33, 95% CI 0.12 to 0.53, moderate-quality evidence, I^2^ = 23%) compared to control. There was no evidence for an effect of resistance training on QOL (n = 373, SMD 0.13, 95% CI -0.15 to 0.42, low-quality evidence, I^2^ = 0%), fatigue (n = 373, SMD -0.08, 95% CI -0.37 to 0.22, low-quality evidence, I^2^ = 20%), physical function (n = 198, SMD 0.61, 95% CI -0.73 to 1.95, very low-quality evidence, I^2^ = 83%), or upper-body strength (n = 413, SMD 0.37, 95% CI -0.07 to 0.80, very low-quality evidence, I^2^ = 69%).

**Conclusions:**

Resistance training may improve lower-body strength in patients undergoing chemotherapy treatment compared to control.

**Supplementary Information:**

The online version contains supplementary material available at 10.1007/s00520-024-08766-y.

## Introduction

The global burden of cancer continues to rise with approximately 19.3 million new cases and nearly 10 million cancer-related deaths recorded in 2020 [1]. Chemotherapy, a common treatment modality for various types of cancer, is administered as both primary and secondary treatment with the aim of eradicating the cancer or inhibiting the proliferation of malignant cells [2]. The use of chemotherapy is predicated on its ability to decrease tumour burden, prevent recurrence and improve survival outcomes [3]. However, despite its undisputed therapeutic benefits, chemotherapy is associated with an array of adverse side effects including impaired physical function, reduced muscular strength, and elevated fatigue [4–6]. These adverse effects reduce patients’ quality of life (QOL), ability to function independently, and negatively impacts psychological wellbeing [7], resulting in the necessity to explore and implement interventions capable of attenuating the negative effects of chemotherapy.

Exercise has been extensively researched and advocated as a strategy to mitigate the adverse side effects associated with chemotherapy [8] and evidence suggests that exercise can be beneficial in counteracting chemotherapy-induced side effects by enhancing QOL [9], reducing fatigue [10], improving physical function [11] and enhancing muscular strength [12]. This evidence has given rise to the development of cancer-specific exercise guidelines, which recommend that people both during and after cancer treatment participate in moderate-intensity aerobic training 2–3 times per week, for 20–30 min, combined with resistance training 2 times per week, using 2 sets of 8–15 repetitions for major muscle groups of moderate to vigorous intensity [8]. Nonetheless, the literature is dominated by studies that focus on aerobic exercise alone or aerobic exercise combined with resistance exercise.

Resistance training can potentially yield a multitude of benefits during chemotherapy treatment [13]. In general populations, resistance training has been shown to improve muscular function through enhanced motor unit recruitment and synchronization, increased firing rates, and reduced inhibitory reflexes which in turn leads to increased muscular strength and coordination [14–16]. Given that chemotherapy can cause diminished muscular strength and physical function, such adaptations could be crucial in mitigating these adverse effects [13]. A review conducted by McGovern et al. [17] examined the effects of resistance training on muscle mass and strength in patients undergoing chemotherapy and/or radiation therapy, showing significant improvement in lean muscle mass and upper-lower body strength. However, this review did not exclusively focus on chemotherapy as a treatment modality and only incorporated supervised exercise interventions. Given the varying side effects associated with different treatment types, it is crucial to investigate single treatment modalities. Furthermore, with the growing prevalence of unsupervised exercise interventions, it is essential to explore both supervised and unsupervised modalities to gain a comprehensive understanding of the overall impact of resistance training during chemotherapy treatment.

To date, no review has synthesized the evidence on the effects of resistance training alone – that is, not in combination with concomitant exercise or mind–body interventions – on QoL, fatigue, physical function, and muscular strength in patients undergoing chemotherapy treatment. Understanding the overall impact of resistance training alone is essential when developing and administering supportive care approaches for people receiving cancer treatment. Pooling the results of individual studies is essential to determine if an intervention has a positive effect. Synthesizing the evidence-base is also needed to identify gaps in knowledge, inform directions for future research, and address discrepancies between studies. This review examined the available literature to assess the effects of resistance training on specific outcomes in cancer patients, to determine whether resistance training alone can be an effective strategy to improve the physical and psychological wellbeing of patients undergoing chemotherapy.

## Methods

This review was prospectively registered on the open science framework (OSF; https://osf.io/yg4jd/) and is reported in accordance with the Preferred Reporting Items for Systematic Reviews and Meta-Analyses (PRISMA) [18]. A PRISMA checklist is provided in Supplementary Material 1. Changes to the pre-registered protocol were minor and are fully documented and justified in Supplementary material 2.

### Search strategy

Authors (JM and LM) systematically searched databases PubMed (NCBI), Cochrane Central Register of Controlled Trials (CENTRAL) (Wiley), CINAHL (EBSCOhost), SCOPUS (Elsevier), and Web of Science (Clarivate), from inception to 18th September 2023. Searches were performed and reported in accordance with PRISMA-S where applicable. A PRISMA-S checklist is provided in Supplementary Material 3 [19]. Standard Boolean operators (AND, OR) were used to combine search terms. The search terms used to identify relevant studies, along with applied filters and limits, are presented in Supplementary Material 4. No publication date restrictions were applied; however, language was restricted to English.

### Inclusion criteria

Original research articles were included in this review if they met the following inclusion criteria: i) the study was a prospective RCT; ii) participants were aged ≥ 18 years; iii) the resistance training intervention lasted ≥ 2 weeks; iv) the resistance training group was compared to a control group that followed usual care only and did not receive a structured exercise intervention; v) patients were actively undergoing at least one dose of any type of chemotherapy for the treatment of any cancer irrespective of intent (e.g. palliative, curative) or sequence (e.g. first-line, induction); vi) measures of QOL, fatigue, physical function, strength, or power, were collected pre- and post-intervention vii) full-text was available in English.

### Exclusion criteria

Studies were excluded if they met any of the following criteria: i) Patients received chemotherapy and/or other treatments modalities such as radiotherapy, immunotherapy, or targeted therapies during the intervention period; ii) the resistance training intervention was combined with other types of exercise or mind–body therapies such as imagery, relaxation, hypnosis, yoga, meditation, tai-chi, qigong, art therapies etc. iii) the control group received an intervention, such as an exercise or nutritional intervention; iv) the intervention group incorporated a concomitant nutritional intervention (e.g., changes to diet or supplementation); v) studies did not include a pre- and post-intervention measure of QOL, fatigue, upper/lower-body strength, power, or physical function; or vi) measures of physical function were subjective. In accordance with Cochrane guidelines, this review excluded quasi-experimental, observational, and cross-over trials due to it being feasible to conduct RCTs to answer the research question in this context [20].

### Outcomes

Outcomes included measures of QOL, fatigue, physical function, upper-body strength, lower-body strength, and muscular power. QOL could be measured with validated questionnaires, such as the European Organization for Research and Treatment of Cancer Quality of Life Questionnaire Core-30 (EORTC QLQ-C30), Functional Assessment of Cancer Therapy-General (FACT-G), the 36-item short-form (SF-36), or QOL subscales such EORTC QLQ-C30 global health status. Fatigue was measured via symptom specific questionnaires such as the Multidimensional Fatigue Inventory (MFI-20), Functional Assessment of Chronic Illness Therapy – Fatigue Scale, or fatigue sub-scales such as the EORTC QLQ-C30 fatigue scale.

Assessments of physical function included measures such as the 30-s sit-to-stand test, timed up and go and 6-min walk test. Muscular strength outcomes included mass lifted in dynamic strength tests in the upper body (e.g., bench press, overhead press, seated row, latissimus pull down and elbow flexion and extension) and lower body (e.g., squat, leg press, leg flexion and extension) and maximum force achieved in isokinetic tests such as elbow flexion/extension, knee flexion/extension and hip flexion/extension. Muscle power outcomes included power produced in dynamic movements such as a countermovement jump or a leg extension on an isokinetic dynamometer.

### Study selection

Upon completion of the searches, all identified studies were collated into a Microsoft Excel spreadsheet (Microsoft Corporation, Redmond, Washington, USA). Reviewers (JM and LM) independently removed duplicates and screened studies against the eligibility criteria. The screening process was carried out in three distinct phases: i) the exclusion of studies after an initial screen of titles and abstracts; ii) a comprehensive review of full text manuscripts; and iii) data availability. Any disagreements between reviews were resolved through discussion and consultation with a third reviewer (RV). Relevant reviews and reference lists and forward citations of included studies and relevant reviews were manually checked to identify additional eligible studies.

### Near misses

A total of nine studies were judged to meet many, but not all, of the eligibility criteria (i.e., ‘near misses’). These ‘near miss’ studies were excluded for three main reasons i) participants received other types of treatment, such as radiotherapy alongside chemotherapy [21–26]; ii) participants participated in combined aerobic and resistance training [27]; iii) studies included an active control group [28, 29]. These studies along with justifications for exclusion are presented in Supplementary Material 5.

### Data extraction

Data items were extracted from each study onto a project-specific data extraction form (Microsoft Excel). Data included: i) authors and year of publication; ii) study design; iii) participant characteristics; v) treatment details; vi) outcome measures; vii) pre- and post-intervention change score data for each outcome measure (mean and standard deviations). In the case of missing data, corresponding authors were contacted on at least two occasions within a 1-month period. Data conversions were not needed to prepare the data for meta-analyses [20]. If RCTs included multiple intervention groups, we only extracted data from the resistance training-only group and the control group. The data extraction process was completed independently by authors (JM and LM) and reviewed by (RV).

### Risk of *Bias*

The Cochrane risk of bias tool for randomised trials (RoB 2) was used to assess the risk of bias for each included outcome within each study [30]. The principal effect of interest was assessed on an intention-to-treat basis. The RoB 2 assesses outcomes through five domains and a series of signalling questions relating to the: i) randomisation process; ii) deviations from intended interventions; iii) missing outcome data; v) measurement of the outcome and vi) selection of the reported result. Assessments of each domain and the overall risk of bias were characterised as ‘low risk of bias’, ‘high risk of bias’, or ‘some concerns’. An overall bias judgment was taken as the least favourable assessment across all domains. Risk of bias due to missing results in a synthesis was explored with Egger’s test of the intercept [31], and by visually inspecting a funnel plot of the treatment effects plotted against their corresponding sampling variance. RoB 2 judgements were made by two independent authors (JM and LM) and disagreements reviewed by (RV).

### Quality of evidence

Quality of evidence for each outcome was assessed using the Grades of Recommendation, Assessment, Development, and Evaluation (GRADE) approach [32]. The GRADE approach classifies evidence quality into four levels ‘high’, ‘moderate’, ‘low’ and ‘very low’. Randomised trials started with a ‘high quality of evidence’ rating which was then assessed and re-graded based on the following factors: i) risk of bias; ii) inconsistency of results; iii) indirectness of evidence; v) imprecision of results and vi) publication bias [33].

Quality of evidence was downgraded by one level if a serious limitation was identified or by two levels if there was deemed to be a very serious limitation. Two reviewers (JM and LM) independently graded the quality of evidence, with any disagreements reviewed by (RV). An overall rating was given to the entire body of evidence, determined by the outcome with the lowest level of quality [34]. The criteria used for evaluating the certainty of the evidence can be open science framework (OSF; https://osf.io/yg4jd/).

### Statistical analysis

All included studies were narratively synthesized. A meta-analysis of standardised mean difference (SMD) between conditions was performed when two or more studies reported the same outcomes. SMDs were calculated by dividing the mean difference by the pooled standard deviation at baseline, where mean differences were calculated by subtracting the mean pre-post change of the intervention group with the mean pre-post change in the control group. Hedges' g corrections were applied to the SMD to adjust for sample bias. Qualitative descriptors used to interpret the strength of the SMDs were based on Cohen’s criteria ( ±): trivial (< 0.2), small (0.2 to 0.49), moderate (0.5 to 0.79), and large (≥ 0.8) [35].

Statistical heterogeneity between studies was assessed with the Chi-squared test (χ2), and the percentage of variability in effect estimates due to heterogeneity as opposed to sampling error was assessed using the I^2^ statistic. Thresholds for the interpretation of I^2^ were in line with Cochrane recommendations: 0–40% (‘might not be important’), 30–60% (‘may represent moderate heterogeneity’), 50–90% (‘may represent substantial heterogeneity’), and 75–100% (‘considerable heterogeneity’) [36]. The importance of the observed I^2^ value was interpreted alongside its 95% Confidence intervals (CIs) and the p-value from the χ2 test [36].

Meta-analyses were conducted using a random effects model and the restricted maximum likelihood method to estimate between-study variance. A random effects model was used to incorporate potential heterogeneity across studies [37]. CIs and test statistics were calculated via a t-distribution restricted maximum likelihood method using the Hartung-Knapp-Sidik-Jonkman (HKSJ) approach. The HKSJ adjustment for random effects meta-analysis results in superior error rates when the number of included studies is small [38]. Studies were weighted according to the inverse of the sampling variance. When meta-analysis includes multiple outcome measure from the same study, effect estimates were nested within studies using a multi-level structure to account for correlated effects [39]. Forest plots were used to visually display the results of individual studies and pooled syntheses.

Statistical analyses were performed using package metafor in R version 4.0.2 (R Foundation for Statistical Computing, Vienna, Austria). Statistical significance was set at p < 0.05. Data are presented as effect estimates with their corresponding 95% CIs. The search results, dataset, and statistical code are available on the OSF repository (https://osf.io/yg4jd/).

## Results

### Study selection

**Fig.1:**
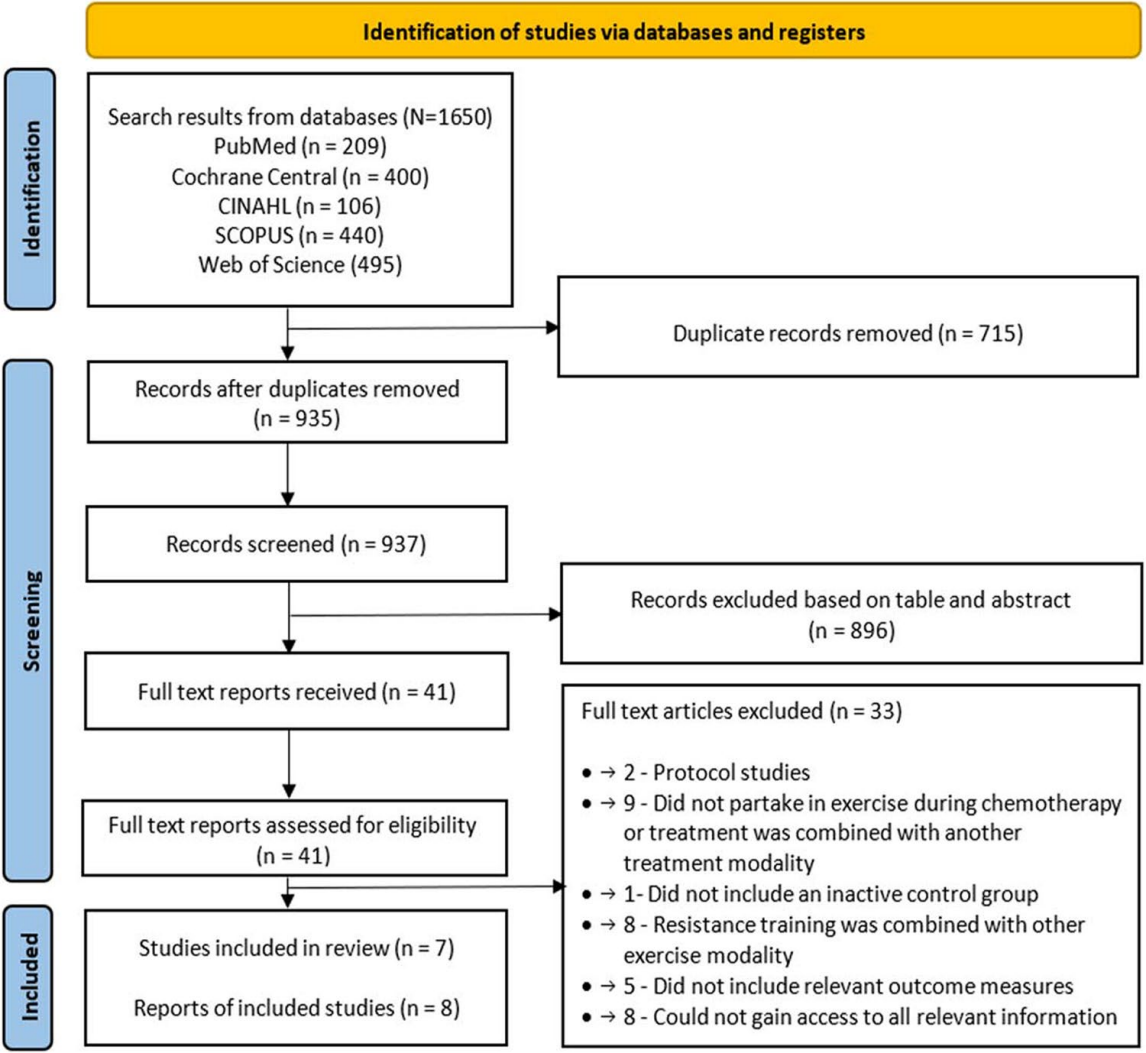
Summary of the study selection process

A total of 1650 records were identified through databases searches, of which 715 were duplicates. Two additional records were identified through citation tracing. After duplications were removed and screening of 937 title and abstracts, 41 full-text articles were assessed for eligibility. A total of 7 RCTs and 8 reports met the eligibility criteria and were included in this review. A summary of the study selection process is presented in Fig. 1.

### Study characteristics

A summary of study characteristics is presented in Table 1. A total of 561 participants were included in this review with 373 included in the QOL and fatigue meta-analyses, 198 included in the physical function meta-analysis, 413 included in the upper-body strength meta-analysis and 555 in the lower-body strength meta-analysis. All studies included in this review compared resistance training to control.

**Table 1:** Summary of study characteristics

Author(s) (year)	Study design	Participant Characteristics	Treatment details	Outcomes Measures relevant to this review
Christensen et al. (2014a) [41] (2014b) [40]	Three-arm RCT CON: Usual care INT: Resistance training REF: Healthy reference group	Male disseminated germ cell cancer patients aged 18–50, no existing cardiovascular or chronic disease CON: N = 15, age 35.8 ± 8.9 INT: N = 15, age 34.4 ± 7.6 HRG: N = 19, age 31.5 ± 6.0	**BEP Therapy:** Cisplatin: 20 mg m^−2^ daily for 5 days Etoposide: 100 mg m^−2^ daily for 5 days Bleomycin: 15000 IE m^−2^ 2 weekly Administered in a 3-week schedule All patients received standard antiemetic treatment during the initial 5 days of each cycle: Prednisolone: 50 mg daily 5HT3-antagonists Metopimazine	EORTC QLQ C30 (Global health status) EORTC QLQ C30 (Fatigue) SF-36 (General health) SF-36 (Fatigue) IKD (MVIC) Knee extensors (Nm)
Courneya et al. (2007) [42]	Three-arm RCT CON: Usual care INT: Resistance training INT: Aerobic exercise training	English- or French-speaking non-pregnant women ≤ 18 years old with stage I to IIIA breast cancer Overall: N = 242, age 49.2 (range 25–78) CON: N = 82, age 49 (range 26–78) INT (RT): N = 82, age 49.5 (range 25–76) INT (AET): N = 78, age 49 (range 30–75)	First line adjuvant chemotherapy Breast conservation surgery **Chemotherapy protocol:** Nontaxane: FEC, AC, CEF Taxane: TAC, AC-Taxane	FACT-An FACT-An (Fatigue) Bench Press 8RM Leg Extension 8RM
Müller et al. (2021) [47]	Three-arm RCT CON: Usual care INT: Resistance training INT: Sensorimotor training	Male and female cancer patients ≥ 18 years of age Overall: N = 163 (M = 25, F = 138), age 53.3 ± 11.5 CON: N = 57, age 54.5 ± 11.9 INT (RT): N = 57, age 53.4 ± 11.7 INT (SMT): N = 49, age 51.7 ± 10.8	Assigned to receive a chemotherapeutic regimen containing at least one of the following agents: - Platinum analogue, e.g. cisplatin, carboplatin, oxaliplatin - Vinca alkaloid, e.g. vincristine - Taxane, e.g. paclitaxel, docetaxel - Suramin - Thalidomide or lenalidomide - Bortezomib	EORTC QLQ C30 (Global health status) EORTC QLQ C30 (Fatigue) IKD (MVIC) Knee extension (Nm)
Schmidt et al. (2015) [43]	Three-arm RCT CON: Usual care INT: Resistance training INT: Endurance training	Female breast cancer patients aged 18–70 Overall: 67CON: N = 26, age 54 ± 11.19 INT (RT): N = 21, age 53 ± 12.55 INT (ET): N = 20, age 56 ± 10.15	Adjuvant chemotherapy without taxane and herceptin	EORTC QLQ C30 (Global health status) EORTC QLQ C30 (Fatigue) MFI-20 Bench Press (Nm) Latissimus pull-down (Nm) Leg Press (Nm)
Schwartz & Winter-stone, (2009) [45]	Three-arm RCT CON: Usual care INT: Resistance training INT: Aerobic exercise training	Female breast, lymphoma, and colon cancer patients ≥ 18 years of age Overall: N = 101, age 47 ± 9.4 (range 27–71) CON: N = 33, age 48 INT (RT): N = 34, age 47 INT (AET): N = 34, age 48	Chemotherapy and steroids (e.g., decadron, prednisone)	12-min walk (m) Overhead press (1RM) Seated row (1RM) Leg extension (1RM)
Schwartz et al., (2007) [46]	Three-arm RCT CON: Usual care INT: Resistance training INT: Aerobic exercise training	Women with invasive breast cancer (stage I to IIIA) Overall: N = 66, CON: N = 23, age 46.26 ± 9.8 INT (RT): N = 21, age 50.1 ± 8.7 INT (AET): N = 22, age 48.32 ± 12.6	Chemotherapy with doxorubicin or methotrexate Cyclophosphamide, methotrexate, and 5-fluorouracil Doxorubicin and cyclophosphamide Doxorubicin, cyclophosphamide, and a taxane Erythropoietin Tamoxifen	12-min walk (m) Overhead press (1RM) Seated row (1RM) Leg extension (1RM)
Wiskemann et al., (2019) [44]	Three-arm RCT CON: Usual care INT: Supervised Resistance training INT: Home-based resistance training	Resectable or non-resectable pancreatic cancer (stages I–IV) Overall: N = 65, CON: N = 22, age 57.8 ± 8.2 INT (SRT): N = 12, age 62.8 ± 6.4 INT (HRT): N = 31, age 61.1 ± 8.7	Primarily adjuvant chemotherapy with some patients receiving neoadjuvant chemotherapy	6-min walk IKD (MIPT) Elbow Flexor (Nm) IKD (MIPT) Elbow Extensor (Nm) IKD (MIPT) Knee Flexor (Nm) IKD (MIPT) Knee Extensor (Nm) IKD (MIPT) Hip Flexor (Nm) IKD (MIPT) Hip Extensor (Nm) IKD (MVIC) Elbow Flexors (Nm) IKD (MVIC) Knee extensors (Nm) IKD (MVIC) Hip Flexors (Nm)

AC = Adriamycin/Cyclophosphamide, AET = Aerobic Training, BEP = Bleomycin, Etoposide And Platinum, CEF = Cyclophosphamide/Epirubicin/5-Fluorouracil, CON = Control Group, EORTC QLQ C30 = European Organisation For Research And Treatment Of Cancer Quality Of Life Questionnaire, ET = Endurance Training, FACT-An = Functional Assessment Of Cancer Therapy-Anaemia, FEC = 5-Fluorouracil/Epirubicin/Cyclophosphamide, HRG = Healthy Reference Group, IKD = Isokinetic Dynamometer, INT = Intervention Group, M = Metres, MFI-20 = Multidimensional Fatigue Inventory, MIPT = Maximal Isokinetic Peak Torque, MVIC = Maximum Voluntary Isometric Contraction, N = Number, Nm = Newton-Meter, RCT = Randomised Control Trial, REF = Reference Group, RM = Repetition Maximum, RT = Resistance Training, SF-36 = 36-Item Short Form Health Survey questionnaire, SMT = Sensorimotor Training, TAC = Docetaxel/Adriamycin/Cyclophosphamide

A summary of intervention characteristics can be found in Supplementary Material 6. Five out of the eight included studies incorporated machine-based interventions [40–44], while three included resistance bands and/or free-weights [44–46] and a single study combined machines with body weight resistance exercises [47]. All interventions were designed to target all major muscle groups of the upper and lower body. Regarding the setting, five studies were supervised [40–44], three home-based [44–46], and one incorporated a combined approach of supervised and home-based exercise [47]. The mean (SD) duration of resistance training intervention was 19 (7.7) weeks. The frequency of interventions ranged from two to four sessions per week and session durations ranged from 20–70 min. Training intensity varied from 50–80% of one repetition maximum or between 14 to 16 on Borg’s 6–20 rating of perceived exertion (RPE) scale [48]. The number of sets performed ranged from 1 to 4, with repetitions between 8 and 20. Exercise intensity was generally progressed by increasing the load after the desired number of sets and reps were achieved. However, two studies increased exercise intensity based on RPE [43, 44]. Adherence to resistance training interventions varied, ranging from 49–84%, with two studies not reporting adherence [43, 46]

### Risk of *Bias*

**Fig.2:**
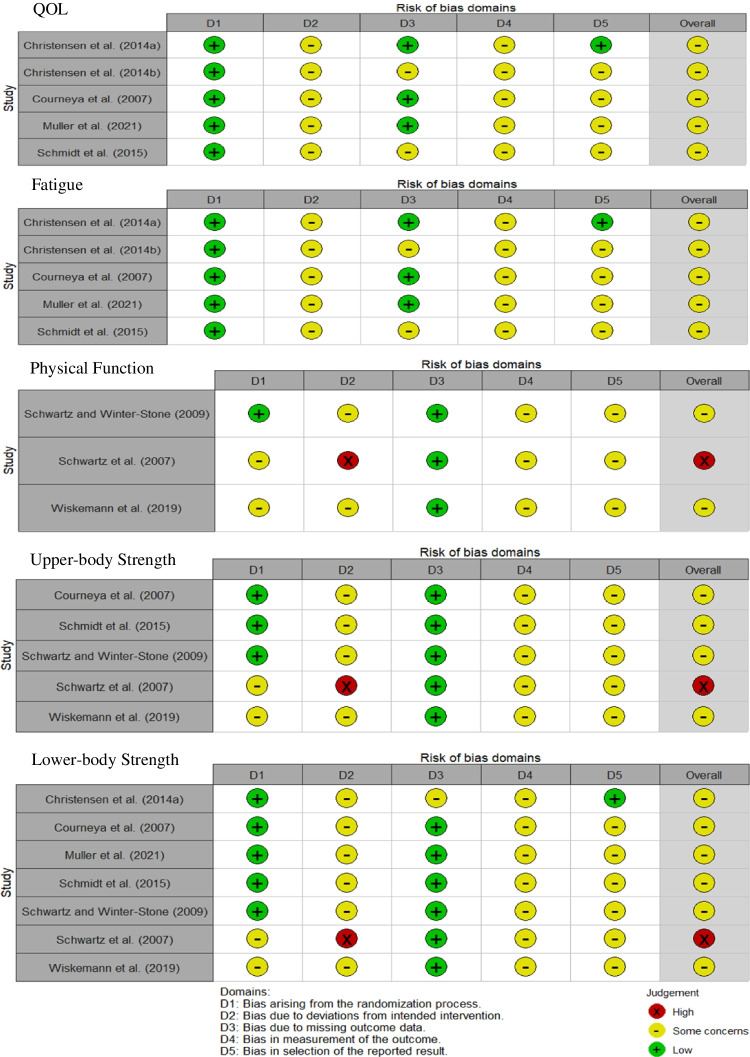
Risk of Bias assessment for all outcomes

Risk of bias was assessed for all outcomes. QOL and fatigue outcomes were assessed as having some concerns due to deviations from the intended intervention, measurement of the outcome and selection of the reporting of results. Physical function, upper-body strength and lower-body strength were assessed as having high risk of bias due to participants deviating from the intended intervention. Funnel plots and Egger’s test indicated that publication bias was not present in upper-body (*p* < 0.26) and lower-body strength (*p* < 0.46). Funnel plots can be found in Supplementary Material 7. A summary of all the results for risk of bias assessments can be seen in Fig. 2 and justifications for grading are available on OSF (https://osf.io/yg4jd/).

### Quality of evidence

The GRADE assessments revealed that the quality of evidence for QOL and fatigue was low. This was attributed to a high risk of bias in one individual study and the indirectness of evidence, with ≥ 50% of the included studies not reporting all components of the FITT principle (frequency, intensity, time, and type). The quality of evidence for physical function was rated as very low due to a high risk of bias, considerable heterogeneity, indirectness of evidence, and imprecision of results, with the 95% CI of the pooled SMD spanning greater than 0.8 and -0.8. The quality of evidence for upper-body strength was very low due to a high risk of bias, substantial heterogeneity, and indirectness of evidence because ≥ 50% of the included studies investigated the same type of cancer. The quality of evidence for lower-body strength was moderate due to a high risk of bias. A summary of findings table and GRADE evidence profile are presented in Table 2.

**Table 2:** Summary of findings and GRADE evidence profile

Summary of findings				Quality assessment					
Outcome	No. of participants (studies)	Pooled SMD (95% CI)	I^2^ (*p value)*	Risk of bias	Inconsistency	Indirectness	Imprecision	Publication bias	Quality rating
Quality of life	373 (5)	0.13 [-0.15, 0.42]	0% (p = 0.40)	Serious limitation (a)	No serious limitation	Serious limitation	No serious limitation	Undetected (b)	Low
Fatigue	373 (5)	-0.08 [-0.37, 0.22]	19.56% (p = 0.36)	Serious limitation (a)	No serious limitation	Serious limitation	No serious limitation	Undetected (b)	Low
Physical function	198 (3)	0.61 [-0.73, 1.95]	83.49% (*p* < 0.001)	Serious limitation (a)	Serious limitations	Serious limitation	Very serious limitation	Undetected (b)	Very low
Upper-body Strength	413 (5)	0.37 [-0.07, 0.80]	68.51% (*p* = 0.007)	Serious limitation (a)	Serious limitations	Serious limitation	No serious limitation	Undetected	Very low
Lower-body Strength	555 (7)	0.33 [0.12, 0.53]	23.19% (*p* = 0.32)	Serious limitation (a)	No serious limitation	No serious limitations	No serious limitation	Undetected	Moderate

95% CI = 95% confidence interval; GRADE = Grading of Recommendations, Assessment, Development and Evaluation; SMD = standardised mean difference

aMore than 50% of studies were judged to have some concerns in three or more domains in the Cochrane risk of bias tool for randomized trials (RoB 2)

bdid not perform a funnel plot or Egger’s test of the intercept analysis because the meta-analysis included less than 10 effect estimates. However, none of the individual effect estimates included in the meta-analysis reached the conventional threshold for statistical significance (i.e. p < 0.05), and therefore publication bias was considered unlikely

### Effects on lower-body strength

**Fig. 3:**
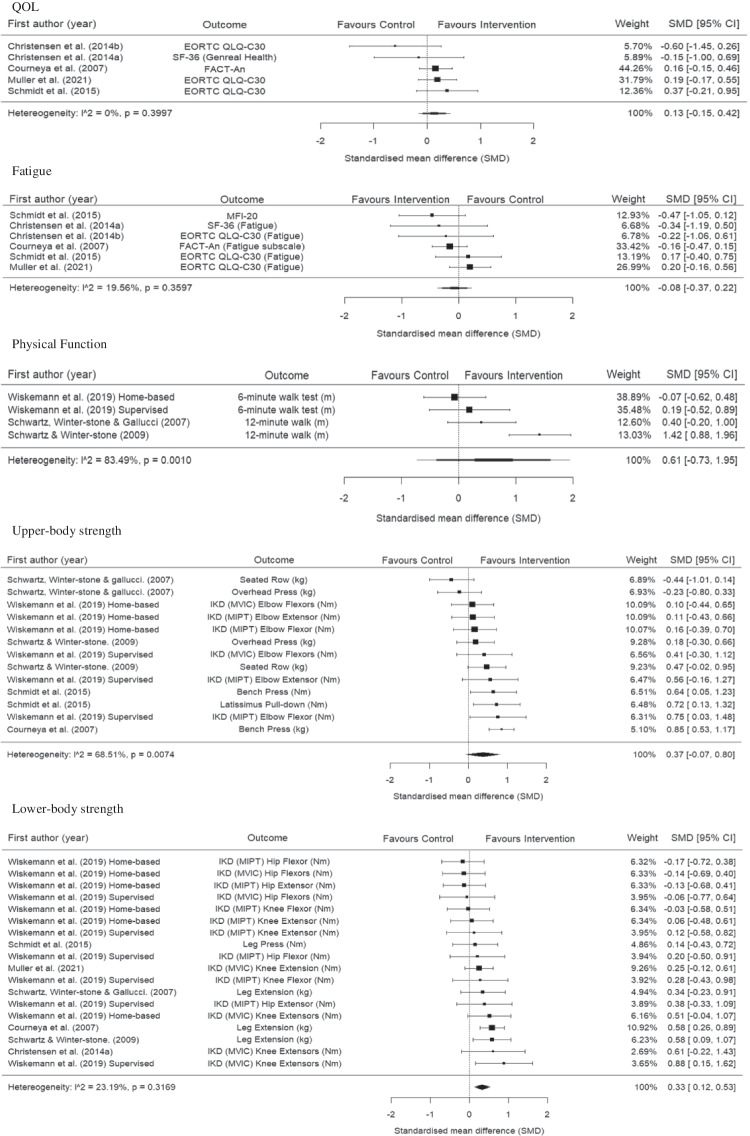
Forest plots of the results from multi-level random-effects meta-analyses on exercise intervention effects on QOL, fatigue, physical function, upper-body and lower-body strength. Data are presented as SMDs between exercise and usual care groups with corresponding 95% confidence intervals (95% CIs). EORTC QLQ-30 = The European Organization for Research and Treatment of Cancer Quality of Life Questionnaire-30, FACT-An = Functional Assessment of Cancer Therapy-Anaemia, IKD = Isokinetic Dynamometer, kg = Kilograms, M = Metres, MFI 20 = Multidimensional Fatigue Inventory, MIPT = Maximal Isokinetic Peak Torque, MVIC = Maximal Voluntary Isometric Contraction, Nm = Newton-metre, SF-36 = 36-Item Short Form Health Survey questionnaire

The meta-analysis for lower-body strength included seven RCTs [41–47] consisting of eighteen effect estimates and 555 participants. The meta-analysis showed that resistance training led to small and significant improvement in lower-body strength (SMD 0.33, 95% CI 0.12 to 0.53; p = 0.004; Fig. 3). There was evidence that heterogeneity might not be important (*I*^2^ = 23.19%, *p* = 0.32).

### Effects on QOL

The meta-analysis for QOL included five RCTs [40–43, 47] consisting of five effect estimates and 373 participants. The meta-analysis showed a trivial non-significant difference between resistance training interventions and usual care on QOL (SMD 0.13, 95% CI − 0.15 to 0.42; p = 0.27; Fig. 3). There was evidence that heterogeneity might not be important (*I*^2^ = 0%, *p* = 0.40).

### Effects on fatigue

The meta-analysis for fatigue included five RCTs [40–43, 47] consisting of six effect estimates and 373 participants. The meta-analysis showed a trivial non-significant difference between resistance training interventions and usual care on fatigue (SMD -0.08, 95% CI − 0.37 to 0.22; p = 0.55; Fig. 3). There was evidence that heterogeneity might not be important (*I*^2^ = 19.56%, *p* = 0.36).

### Effects on physical function

The meta-analysis for physical function included three RCTs [44–46] consisting of four effect estimates and 198 participants. The meta-analysis showed a medium SMD but non-significant difference between resistance training interventions and usual care on physical function (SMD 0.61, 95% CI − 0.73 to 1.95; p = 0.24; Fig. 3). There was evidence of substantial heterogeneity (*I*^2^ = 83.49%, *p* < 0.001).

### Effects on upper-body strength

The meta-analysis for upper-body strength included five RCTs [42–46] consisting of thirteen effect estimates and 413 participants. The meta-analysis showed a small non-significant difference between resistance training interventions and usual care on upper-body strength (SMD 0.37, 95% CI − 0.07 to 0.80; p = 0.089; Fig. 3). There was evidence of substantial heterogeneity (*I*^2^ = 68.51%, *p* < 0.007).

## Discussion

This is the first systematic review and meta-analysis to investigate the effects of resistance training on QOL, fatigue, physical function, and muscular strength during chemotherapy. Our findings demonstrate that resistance has the potential to significantly increase lower-body strength during chemotherapy. By contrast, there was no evidence for an effect of resistance training on other outcomes including QOL, fatigue, physical function, and upper-body strength.

In line with our findings, a previous meta-analysis of nine studies reported that resistance training improves lower-body strength in people undergoing adjuvant chemotherapy and/or radiation therapy for cancer, with a standardized mean difference (SMD) of 0.58 (95% CI 0.18 to 0.98) [17]. However, this review also showed a considerably higher unexplained heterogeneity between studies (I^2^ = 91%) compared to our review (I^2^ = 23.19%), suggesting a lower quality of evidence. It additionally reported a significant increase in upper-body strength, which contrasts with our findings. The differences between these findings could be attributed to several factors. Firstly, our review focused solely on chemotherapy treatments and excluded radiation therapy, making our inclusion criteria more restrictive. Secondly, the smaller sample size in our review may have led to less precise effect estimates. Thirdly, the previous review involved heavier loads (60–80% of 1RM for 2–3 sets of 5–12 reps), which are known to yield greater strength gains, compared to the lighter loads (50–70% of 1RM for 1–3 sets of 8–20 reps) used in our review [49]. Furthermore, while our review included studies that used resistance bands, the previous review exclusively used machine-based exercises. This is significant as the principle of specificity suggests that outcomes are optimised when training methods closely match assessment conditions [50]. Therefore, machine-based assessments are likely to benefit more from machine-based interventions due to the similarity in both equipment and movement patterns. Lastly, the level of supervision varied; our review included both supervised and unsupervised interventions, in contrast to the previous review where all studies were supervised. Supervised exercise is well-known to lead to superior adaptations in cancer survivors [8].

Strengths of this review include the intentional exclusion of active control groups, which ostensibly enhanced our ability to detect the true effect of resistance training compared to control. This methodological decision, despite limiting the number of included RCTs, was essential for quantifying the independent effects of resistance training, thus ensuring a more precise understanding of its effects. Furthermore, this review incorporated a broad spectrum of exercise programmes, including supervised, unsupervised, and mixed-method approaches. This offered a broader examination of the overall effects of resistance training on individuals undergoing chemotherapy treatment, including pragmatic protocols that may be more likely to be implementable within healthcare systems. To further support transparency and replicability, the protocol, analysis plan, search results, and statistical code are publicly available on OSF (https://osf.io/yg4jd/).

This review has some limitations. Only eight RCTs, were eligible for this review, partly due to our eligibility criteria excluded studies with 'active' control groups, including other types of exercise or mind–body therapies. QOL and fatigue outcomes were judged to have some risk of bias concerns due to the lack of blinding of participants and assessors, and the absence of preregistered study protocols and analysis plans. A lack of blinding can cause biased reporting particularly on subjective outcomes where participants' perceptions could be influenced by their expectations about an intervention's effectiveness. Furthermore, the absence of these preregistered protocols raises concerns about the potential for selective reporting [51]. Additionally, physical function, upper-body strength, and lower-body strength were judged as having a high risk of bias due to deviations in the intended interventions, with participants reporting participation in aerobic exercise [46]. Such deviations could have affected the results, making it difficult to attribute any observed effects solely to the resistance training intervention being studied. Overall, the quality of evidence for QOL and fatigue was low. This was attributed to high risk of bias and the indirectness of evidence, indicating serious limitations due to not reporting all components of the FITT principle, specifically session duration [40–42, 46]. Not reporting these components can lead to a lack of understanding surrounding the context and specifics of the intervention which can lead to challenges in interpreting the results or replicating the study in future trials. The quality of evidence for physical function was deemed very low due to imprecise results showing wide confidence intervals (SMD 0.61, 95% CI − 0.73 to 1.95). Wide confidence intervals reflect greater variability in the data, meaning that the true effect of the intervention is unclear [52]. The lack of significant heterogeneity for QOL, fatigue, and lower-body strength suggests that the results and outcome measures used were consistent across different studies. However, there was high heterogeneity for physical function and substantial heterogeneity for upper-body strength likely due to sampling errors associated with small sample sizes and varied outcome measures included in the analysis. This review focused solely on the benefits of resistance training during chemotherapy treatment and did not assess the potential harms. The risks of exercising during chemotherapy are currently unknown [53]; therefore, caution is warranted when balancing the benefits against possible adverse effects. Finally, the literature search was restricted to full-text manuscripts available in English and may therefore have missed some relevant studies written in other languages.

Future research should focus on several key areas to enhance the understanding and effectiveness of resistance training on chemotherapy-related side effects. Firstly, more research is needed exploring the isolated effects of resistance training during chemotherapy treatment as research is limited in this area. To improve the quality of evidence, it is essential that future studies adhere to published guidelines for accurately reporting exercise interventions [54]. This will ensure clarity and consistency in how interventions are implemented and evaluated. Additionally, standardising strength assessments is crucial for reducing potentially inflated heterogeneity, which can skew the overall understanding of resistance training effectiveness. Although challenging, another vital step is to blind outcome assessors and data analysts to minimise bias in measuring outcomes [55]. Finally, prospective trial registration and promoting open access are important for transparency and reproducibility, reducing risk of bias and enhancing the overall quality of evidence [56, 57].

This review showed that resistance training during chemotherapy may leads to significant improvements in lower-body strength when compared to usual care. By contrast, there is no evidence that resistance training during chemotherapy improves QOL, fatigue, physical function, and upper-body strength. The overall body of evidence was judged to be of very low quality due to the risk of bias, indirectness of evidence, and imprecision of results therefore results should be interpreted with caution. Future RCTs should focus on improving the quality and body of evidence in this area.

## Supplementary Information

Below is the link to the electronic supplementary material.

Supplementary file1 (DOCX 115 KB)

## Data Availability

All data analysed during the meta-analyses, and code used, is available on the Open Science Framework (https://osf.io/yg4jd/).

## References

[1] Sung H, Ferlay J, Siegel RL et al (2021) Global cancer statistics 2020: GLOBOCAN estimates of incidence and mortality worldwide for 36 cancers in 185 countries. CA Cancer J Clin 71:209–249. 10.3322/caac.21660 33538338 10.3322/caac.21660

[2] Anand U, Dey A, Chandel AKS et al (2022) Cancer chemotherapy and beyond: current status, drug candidates, associated risks and progress in targeted therapeutics. Genes Dis 10:1367–1401. 10.1016/j.gendis.2022.02.007 37397557 10.1016/j.gendis.2022.02.007 PMC10310991

[3] Moo T-A, Sanford R, Dang C, Morrow M (2018) Overview of breast cancer therapy. PET Clin 13:339–354. 10.1016/j.cpet.2018.02.006 30100074 10.1016/j.cpet.2018.02.006 PMC6092031

[4] Bower JE (2014) Cancer-related fatigue—mechanisms, risk factors, and treatments. Nat Rev Clin Oncol 11:597–609. 10.1038/nrclinonc.2014.127 25113839 10.1038/nrclinonc.2014.127 PMC4664449

[5] Klassen O, Schmidt ME, Ulrich CM et al (2017) Muscle strength in breast cancer patients receiving different treatment regimes. J Cachexia Sarcopenia Muscle 8:305–316. 10.1002/jcsm.12165 27896952 10.1002/jcsm.12165 PMC5377413

[6] Lewandowska A, Rudzki G, Lewandowski T et al (2020) Quality of life of cancer patients treated with chemotherapy. Int J Environ Res Public Health 17:6938. 10.3390/ijerph17196938 32977386 10.3390/ijerph17196938 PMC7579212

[7] Stein KD, Syrjala KL, Andrykowski MA (2008) Physical and psychological long-term and late effects of cancer. Cancer 112:2577–2592. 10.1002/cncr.23448 18428205 10.1002/cncr.23448 PMC7047657

[8] Campbell KL, Winters-Stone K, Wiskemann J et al (2019) Exercise guidelines for cancer survivors: consensus statement from international multidisciplinary roundtable. Med Sci Sports Exerc 51:2375–2390. 10.1249/MSS.0000000000002116 31626055 10.1249/MSS.0000000000002116 PMC8576825

[9] Fukushima T, Nakano J, Hashizume K et al (2021) Effects of aerobic, resistance, and mixed exercises on quality of life in patients with cancer: a systematic review and meta-analysis. Complement Ther Clin Pract 42:101290. 10.1016/j.ctcp.2020.101290 33360071 10.1016/j.ctcp.2020.101290

[10] Medeiros Torres D, Jorge Koifman R, da Silva SS (2022) Impact on fatigue of different types of physical exercise during adjuvant chemotherapy and radiotherapy in breast cancer: systematic review and meta-analysis. Support Care Cancer Off J Multinatl Assoc Support Care Cancer 30:4651–4662. 10.1007/s00520-022-06809-w 10.1007/s00520-022-06809-w 35064331

[11] Sweegers MG, Altenburg TM, Chinapaw MJ et al (2018) Which exercise prescriptions improve quality of life and physical function in patients with cancer during and following treatment? a systematic review and meta-analysis of randomised controlled trials. Br J Sports Med 52:505–513. 10.1136/bjsports-2017-097891 28954800 10.1136/bjsports-2017-097891

[12] Padilha CS, Marinello PC, Galvão DA et al (2017) Evaluation of resistance training to improve muscular strength and body composition in cancer patients undergoing neoadjuvant and adjuvant therapy: a meta-analysis. J Cancer Surviv 11:339–349. 10.1007/s11764-016-0592-x 28054255 10.1007/s11764-016-0592-x

[13] Champ CE, Carpenter DJ, Diaz AK et al (2023) Resistance training for patients with cancer: a conceptual framework for maximizing strength, power, functional mobility, and body composition to optimize health and outcomes. Sports Med 53:75–89. 10.1007/s40279-022-01759-z 36175646 10.1007/s40279-022-01759-z

[14] Carroll TJ, Riek S, Carson RG (2001) Neural adaptations to resistance training. Sports Med 31:829–840. 10.2165/00007256-200131120-00001 11665911 10.2165/00007256-200131120-00001

[15] Jenkins NDM, Miramonti AA, Hill EC, et al (2017) Greater Neural Adaptations following High- vs. Low-Load Resistance Training. Front Physiol 8. 10.3389/fphys.2017.00331 10.3389/fphys.2017.00331 PMC5447067 28611677

[16] Walker S (2021) Evidence of resistance training-induced neural adaptation in older adults. Exp Gerontol 151:111408. 10.1016/j.exger.2021.111408 34022275 10.1016/j.exger.2021.111408

[17] McGovern A, Mahony N, Mockler D, Fleming N (2022) Efficacy of resistance training during adjuvant chemotherapy and radiation therapy in cancer care: a systematic review and meta-analysis. Support Care Cancer 30:3701–3719. 10.1007/s00520-021-06708-6 34993651 10.1007/s00520-021-06708-6

[18] Page MJ, McKenzie JE, Bossuyt PM et al (2021) The PRISMA 2020 statement: an updated guideline for reporting systematic reviews. BMJ 372:n71. 10.1136/bmj.n71 33782057 10.1136/bmj.n71 PMC8005924

[19] Rethlefsen ML, Kirtley S, Waffenschmidt S et al (2021) PRISMA-S: an extension to the PRISMA statement for reporting literature searches in systematic reviews. Syst Rev 10:39. 10.1186/s13643-020-01542-z 33499930 10.1186/s13643-020-01542-z PMC7839230

[20] McKenzie JE, Brennan SE, Ryan RE, Thomson HJ, Johnston RV, Thomas J (2023) Chapter 3: Defining the criteria for including studies and how they will be grouped for the synthesis. In: Higgins JPT, Thomas J, Chandler J, Cumpston M, Li T, Page MJ, Welch VA (eds) Cochrane Handbook for Systematic Reviews of Interventions version 6.4 (updated August 2023). Cochrane. Available from www.training.cochrane.org/handbook

[21] Cešeiko R, Eglītis J, Srebnijs A et al (2019) The impact of maximal strength training on quality of life among women with breast cancer undergoing treatment. Exp Oncol 41:166–172. 10.32471/exp-oncology.2312-8852.vol-41-no-2.13249 31262153 10.32471/exp-oncology.2312-8852.vol-41-no-2.13249

[22] Cešeiko R, Thomsen SN, Tomsone S et al (2020) Heavy resistance training in breast cancer patients undergoing adjuvant therapy. Med Sci Sports Exerc 52:1239–1247. 10.1249/MSS.0000000000002260 31876673 10.1249/MSS.0000000000002260

[23] Cheng D, Wang X, Hu J et al (2021) Effect of Tai Chi and resistance training on cancer-related fatigue and quality of life in middle-aged and elderly cancer patients. Chin J Integr Med 27:265–272. 10.1007/s11655-021-3278-9 33420583 10.1007/s11655-021-3278-9

[24] Eisenhut L, Sadeghi-Bahmani D, Gerber M et al (2022) Effects of two types of exercise training on psychological well-being, sleep and physical fitness in patients with high-grade glioma (WHO III and IV). J Psychiatr Res 151:354–364. 10.1016/j.jpsychires.2022.03.058 35537372 10.1016/j.jpsychires.2022.03.058

[25] Hu Q, Zhao D (2021) Effects of resistance exercise on complications, cancer-related fatigue and quality of life in nasopharyngeal carcinoma patients undergoing chemoradiotherapy: A randomised controlled trial. Eur J Cancer Care (Engl) 30:e13355. 10.1111/ecc.13355 33159422 10.1111/ecc.13355

[26] Kilbreath SL, Refshauge KM, Beith JM et al (2012) Upper limb progressive resistance training and stretching exercises following surgery for early breast cancer: a randomized controlled trial. Breast Cancer Res Treat 133:667–676. 10.1007/s10549-012-1964-1 22286332 10.1007/s10549-012-1964-1

[27] Loh KP, Kleckner IR, Lin P-J et al (2019) Effects of a home-based exercise program on anxiety and mood disturbances in older adults with cancer receiving chemotherapy. J Am Geriatr Soc 67:1005–1011. 10.1111/jgs.15951 31034591 10.1111/jgs.15951 PMC6544022

[28] Schmidt ME, Wiskemann J, Armbrust P et al (2015) Effects of resistance exercise on fatigue and quality of life in breast cancer patients undergoing adjuvant chemotherapy: a randomized controlled trial. Int J Cancer 137:471–480. 10.1002/ijc.29383 25484317 10.1002/ijc.29383

[29] Wehrle A, Kneis S, Dickhuth H-H et al (2019) Endurance and resistance training in patients with acute leukemia undergoing induction chemotherapy—a randomized pilot study. Support Care Cancer 27:1071–1079. 10.1007/s00520-018-4396-6 30121789 10.1007/s00520-018-4396-6

[30] Higgins JPT, Savović J, Page MJ, Elbers RG, Sterne JAC (2023) Chapter 8: Assessing risk of bias in a randomized trial. In: Higgins JPT, Thomas J, Chandler J, Cumpston M, Li T, Page MJ, Welch VA (eds) Cochrane Handbook for Systematic Reviews of Interventions version 6.4 (updated August 2023). Cochrane. Available from www.training.cochrane.org/handbook

[31] Egger M, Smith GD, Schneider M, Minder C (1997) Bias in meta-analysis detected by a simple, graphical test. BMJ 315:629–634. 10.1136/bmj.315.7109.629 9310563 10.1136/bmj.315.7109.629 PMC2127453

[32] Guyatt GH, Oxman AD, Vist GE et al (2008) GRADE: an emerging consensus on rating quality of evidence and strength of recommendations. BMJ 336:924–926. 10.1136/bmj.39489.470347.AD 18436948 10.1136/bmj.39489.470347.AD PMC2335261

[33] Schünemann HJ, Higgins JPT, Vist GE, Glasziou P, Akl EA, Skoetz N, Guyatt GH (2023) Chapter 14: Completing ‘Summary of findings’ tables and grading the certainty of the evidence. In: Higgins JPT, Thomas J, Chandler J, Cumpston M, Li T, Page MJ, Welch VA (eds) Cochrane Handbook for Systematic Reviews of Interventions version 6.4 (updated August 2023). Cochrane. Available from www.training.cochrane.org/handbook

[34] Guyatt G, Oxman AD, Sultan S et al (2013) GRADE guidelines: 11. Making an overall rating of confidence in effect estimates for a single outcome and for all outcomes. J Clin Epidemiol 66:151–157. 10.1016/j.jclinepi.2012.01.006 22542023 10.1016/j.jclinepi.2012.01.006

[35] Cohen J (1988) Statistical Power Analysis for the Behavioral Sciences, 2nd edn. Routledge, New York

[36] Deeks JJ, Higgins JPT, Altman DG (eds) (2023) Chapter 10: Analysing data and undertaking meta-analyses. In: Higgins JPT, Thomas J, Chandler J, Cumpston M, Li T, Page MJ, Welch VA (eds) Cochrane Handbook for Systematic Reviews of Interventions version 6.4 (updated August 2023). Cochrane. Available from www.training.cochrane.org/handbook

[37] Orange ST, Hritz A, Pearson L et al (2022) Comparison of the effects of velocity-based vs. traditional resistance training methods on adaptations in strength, power, and sprint speed: a systematic review, meta-analysis, and quality of evidence appraisal. J Sports Sci 40:1220–1234. 10.1080/02640414.2022.2059320 35380511 10.1080/02640414.2022.2059320

[38] IntHout J, Ioannidis JP, Borm GF (2014) The Hartung-Knapp-Sidik-Jonkman method for random effects meta-analysis is straightforward and considerably outperforms the standard DerSimonian-Laird method. BMC Med Res Methodol 14:25. 10.1186/1471-2288-14-25 24548571 10.1186/1471-2288-14-25 PMC4015721

[39] Van den Noortgate W, López-López JA, Marín-Martínez F, Sánchez-Meca J (2013) Three-level meta-analysis of dependent effect sizes. Behav Res Methods 45:576–594. 10.3758/s13428-012-0261-6 23055166 10.3758/s13428-012-0261-6

[40] Christensen JF, Tolver A, Andersen JL et al (2014) Resistance training does not protect against increases in plasma cytokine levels among germ cell cancer patients during and after chemotherapy. J Clin Endocrinol Metab 99:2967–2976. 10.1210/jc.2013-4495 25050898 10.1210/jc.2013-4495

[41] Christensen JF, Jones LW, Tolver A et al (2014) Safety and efficacy of resistance training in germ cell cancer patients undergoing chemotherapy: a randomized controlled trial. Br J Cancer 111:8–16. 10.1038/bjc.2014.273 24867693 10.1038/bjc.2014.273 PMC4090736

[42] Courneya KS, Segal RJ, Mackey JR et al (2007) Effects of aerobic and resistance exercise in breast cancer patients receiving adjuvant chemotherapy: a multicenter randomized controlled Trial. J Clin Oncol 25:4396–4404. 10.1200/JCO.2006.08.2024 17785708 10.1200/JCO.2006.08.2024

[43] Schmidt T, Weisser B, Dürkop J et al (2015) Comparing endurance and resistance training with standard care during chemotherapy for patients with primary breast cancer. Anticancer Res 35(10):5623–5630s 26408735

[44] Wiskemann J, Clauss D, Tjaden C et al (2019) Progressive resistance training to impact physical fitness and body weight in pancreatic cancer patients: a randomized controlled trial. Pancreas 48:257–266. 10.1097/MPA.0000000000001221 30589829 10.1097/MPA.0000000000001221

[45] Schwartz AL, Winters-Stone K (2009) Effects of a 12-month randomized controlled trial of aerobic or resistance exercise during and following cancer treatment in women. Phys Sportsmed 37:62–67. 10.3810/psm.2009.10.1730 20048529 10.3810/psm.2009.10.1730

[46] Schwartz AL, Winters-Stone K, Gallucci B (2007) Exercise effects on bone mineral density in women with breast cancer receiving adjuvant chemotherapy. Oncol Nurs Forum 34:627–633. 10.1188/07.ONF.627-633 17573321 10.1188/07.ONF.627-633

[47] Müller J, Weiler M, Schneeweiss A et al (2021) Preventive effect of sensorimotor exercise and resistance training on chemotherapy-induced peripheral neuropathy: a randomised-controlled trial. Br J Cancer 125:955–965. 10.1038/s41416-021-01471-1 34226683 10.1038/s41416-021-01471-1 PMC8476560

[48] Borg G (1998) Borg’s perceived exertion and pain scales. Human Kinetics, Champaign, IL, US

[49] Schoenfeld BJ, Grgic J, Ogborn D, Krieger JW (2017) Strength and hypertrophy adaptations between low- vs. high-load resistance training: a systematic review and meta-analysis. J Strength Cond Res 31:3508. 10.1519/JSC.0000000000002200 28834797 10.1519/JSC.0000000000002200

[50] Liguori G, Medicine (ACSM) AC of S (2020) ACSM’s guidelines for exercise testing and prescription. Lippincott Williams & Wilkins

[51] Singh B, Fairman CM, Christensen JF, et al (2021) Outcome reporting bias in exercise oncology trials (OREO): a cross-sectional study. 03.12.21253378

[52] Schünemann HJ, Vist GE, Higgins JPT, Santesso N, Deeks JJ, Glasziou P, Akl EA, Guyatt GH (2023) Chapter 15: Interpreting results and drawing conclusions. In: Higgins JPT, Thomas J, Chandler J, Cumpston M, Li T, Page MJ MJ, Welch VA (eds) Cochrane Handbook for Systematic Reviews of Interventions version 6.4 (updated August 2023). Cochrane. Available from www.training.cochrane.org/handbook

[53] Thomsen SN, Lahart IM, Thomsen LM et al (2023) Harms of exercise training in patients with cancer undergoing systemic treatment: a systematic review and meta-analysis of published and unpublished controlled trials. ClinicalMedicine 59:101937. 10.1016/j.eclinm.2023.101937 10.1016/j.eclinm.2023.101937 PMC10121410 37096190

[54] Slade SC, Dionne CE, Underwood M, Buchbinder R (2016) Consensus on exercise reporting template (CERT): explanation and elaboration statement. Br J Sports Med 50:1428–1437. 10.1136/bjsports-2016-096651 27707738 10.1136/bjsports-2016-096651

[55] Hecksteden A, Faude O, Meyer T, Donath L (2018) How to construct, conduct and analyze an exercise training study? Front Physiol 9. 10.3389/fphys.2018.01007 10.3389/fphys.2018.01007 PMC6094975 30140237

[56] De Angelis C, Drazen JM, Frizelle FA et al (2004) Clinical trial registration: a statement from the international committee of medical journal editors. Ann Intern Med 141:477–478. 10.7326/0003-4819-141-6-200409210-00109 15355883 10.7326/0003-4819-141-6-200409210-00109

[57] Foster ED, Deardorff A (2017) Open science framework (OSF). J Med Libr Assoc JMLA 105:203–206. 10.5195/jmla.2017.88

