# Supplementary material for: Effects of resistance training on quality of life, fatigue, physical function, and muscular strength during chemotherapy treatment: a systematic review and meta-analysis
Source: Support Care Cancer. 2024 Aug 17;32(9):593. doi: 10.1007/s00520-024-08766-y (PMC11330399; doi:10.1007/s00520-024-08766-y)
Supplement: Supplementary file 1 — Supplementary file1 (DOCX 115 KB) [file 520_2024_8766_MOESM1_ESM.docx]

**Supportive Care in Cancer**

**Effects of resistance training on** **quality of life, fatigue, physical function, and muscular strength during chemotherapy treatment: a systematic review and meta-analysis.**

James W Metcalfe^1^*, Samuel T Orange^2,3^, Leigh A Madden^4^, Phil Marshall^1^ and Rebecca V Vince^1^

^1^School of Sport, Exercise & Rehabilitation Sciences, Faculty of Health Sciences, University of Hull, Hull, United Kingdom.

^2^School of Biomedical, Nutritional and Sport Sciences, Faculty of Medical Sciences, Newcastle University, Newcastle upon Tyne, United Kingdom.

^3^Newcastle University Centre for Cancer, Newcastle University, Newcastle upon Tyne, United Kingdom

^4^Centre for Biomedicine, Hull York Medical School, University of Hull, Hull, United Kingdom.

Corresponding author

James W Metcalfe

Email: [Jamesmetcalfe1993@gmail.com](mailto:Jamesmetcalfe1993@gmail.com)

Phone: 07864138445

| **Supplementary Material 1. PRISMA Checklist** | | | |
| --- | --- | --- | --- |
| **Section and Topic** | **Item #** | **Checklist item** | **Location where item is reported** |
| **TITLE** | | |  |
| Title | 1 | Identify the report as a systematic review. | Manuscripts page 1 |
| **ABSTRACT** | | |  |
| Abstract | 2 | See the PRISMA 2020 for Abstracts checklist. | Manuscripts page 2 |
| **INTRODUCTION** | | |  |
| Rationale | 3 | Describe the rationale for the review in the context of existing knowledge. | Manuscripts page 3-4 |
| Objectives | 4 | Provide an explicit statement of the objective(s) or question(s) the review addresses. | Manuscripts page 4 |
| **METHODS** | | |  |
| Eligibility criteria | 5 | Specify the inclusion and exclusion criteria for the review and how studies were grouped for the syntheses. | Manuscripts page 5 |
| Information sources | 6 | Specify all databases, registers, websites, organisations, reference lists and other sources searched or consulted to identify studies. Specify the date when each source was last searched or consulted. | OSF repository (<https://osf.io/yg4jd/>)  (Search Results) |
| Search strategy | 7 | Present the full search strategies for all databases, registers, and websites, including any filters and limits used. | Supplementary material 1 |
| Selection process | 8 | Specify the methods used to decide whether a study met the inclusion criteria of the review, including how many reviewers screened each record and each report retrieved, whether they worked independently, and if applicable, details of automation tools used in the process. | Manuscript page 6 and OSF repository (<https://osf.io/yg4jd/>)  (Search Results) |
| Data collection process | 9 | Specify the methods used to collect data from reports, including how many reviewers collected data from each report, whether they worked independently, any processes for obtaining or confirming data from study investigators, and if applicable, details of automation tools used in the process. | Manuscript page 6 and OSF repository (<https://osf.io/yg4jd/>)  (Search Results) |
| Data items | 10a | List and define all outcomes for which data were sought. Specify whether all results that were compatible with each outcome domain in each study were sought (e.g. for all measures, time points, analyses), and if not, the methods used to decide which results to collect. | Manuscript page 5-6 |
|  | 10b | List and define all other variables for which data were sought (e.g. participant and intervention characteristics, funding sources). Describe any assumptions made about any missing or unclear information. | Manuscript page 10-13 |
| Study risk of bias assessment | 11 | Specify the methods used to assess risk of bias in the included studies, including details of the tool(s) used, how many reviewers assessed each study and whether they worked independently, and if applicable, details of automation tools used in the process. | Manuscript page 7 |
| Effect measures | 12 | Specify for each outcome the effect measure(s) (e.g. risk ratio, mean difference) used in the synthesis or presentation of results. | Manuscript page 8 |
| Synthesis methods | 13a | Describe the processes used to decide which studies were eligible for each synthesis (e.g. tabulating the study intervention characteristics and comparing against the planned groups for each synthesis (item #5)). | Manuscript page 8 |
|  | 13b | Describe any methods required to prepare the data for presentation or synthesis, such as handling of missing summary statistics, or data conversions. | Manuscript page 6 |
|  | 13c | Describe any methods used to tabulate or visually display results of individual studies and syntheses. | Manuscript page 8 |
|  | 13d | Describe any methods used to synthesize results and provide a rationale for the choice(s). If meta-analysis was performed, describe the model(s), method(s) to identify the presence and extent of statistical heterogeneity, and software package(s) used. | Manuscript page 8 |
|  | 13e | Describe any methods used to explore possible causes of heterogeneity among study results (e.g. subgroup analysis, meta-regression). | NA |
|  | 13f | Describe any sensitivity analyses conducted to assess robustness of the synthesized results. | NA |
| Reporting bias assessment | 14 | Describe any methods used to assess risk of bias due to missing results in a synthesis (arising from reporting biases). | Manuscript page 7 |
| Certainty assessment | 15 | Describe any methods used to assess certainty (or confidence) in the body of evidence for an outcome. | Manuscript page 7 |
| **RESULTS** | | |  |
| Study selection | 16a | Describe the results of the search and selection process, from the number of records identified in the search to the number of studies included in the review, ideally using a flow diagram. | Manuscript page 9 |
|  | 16b | Cite studies that might appear to meet the inclusion criteria, but which were excluded, and explain why they were excluded. | OSF repository (<https://osf.io/yg4jd/>)  (Search Results) |
| Study characteristics | 17 | Cite each included study and present its characteristics. | Manuscript page 10-13 and Supplementary material page 4-7 |
| Risk of bias in studies | 18 | Present assessments of risk of bias for each included study. | Manuscript page 14 and Supplementary material page 8 |
| Results of individual studies | 19 | For all outcomes, present, for each study: (a) summary statistics for each group (where appropriate) and (b) an effect estimate and its precision (e.g. confidence/credible interval), ideally using structured tables or plots. | Manuscript page 15 – 17 and Supplementary material page 9 |
| Results of syntheses | 20a | For each synthesis, briefly summarise the characteristics and risk of bias among contributing studies. | Manuscript page 14 and  OSF repository (<https://osf.io/yg4jd/>)  (Risk of bias Justifications) |
|  | 20b | Present results of all statistical syntheses conducted. If meta-analysis was done, present for each the summary estimate and its precision (e.g. confidence/credible interval) and measures of statistical heterogeneity. If comparing groups, describe the direction of the effect. | Manuscript page 15 |
|  | 20c | Present results of all investigations of possible causes of heterogeneity among study results. | NA |
|  | 20d | Present results of all sensitivity analyses conducted to assess the robustness of the synthesized results. | NA |
| Reporting biases | 21 | Present assessments of risk of bias due to missing results (arising from reporting biases) for each synthesis assessed. | Manuscript page 14 & Supplementary material 4 |
| Certainty of evidence | 22 | Present assessments of certainty (or confidence) in the body of evidence for each outcome assessed. | Manuscript page 14-15 |
| **DISCUSSION** | | |  |
| Discussion | 23a | Provide a general interpretation of the results in the context of other evidence. | Manuscript page 18 |
|  | 23b | Discuss any limitations of the evidence included in the review. | Manuscript page 20 |
|  | 23c | Discuss any limitations of the review processes used. | Manuscript page 20-21 |
|  | 23d | Discuss implications of the results for practice, policy, and future research. | Manuscript page 22 |
| **OTHER INFORMATION** | | |  |
| Registration and protocol | 24a | Provide registration information for the review, including register name and registration number, or state that the review was not registered. | Manuscript page 4 |
|  | 24b | Indicate where the review protocol can be accessed, or state that a protocol was not prepared. | Manuscript page 4 |
|  | 24c | Describe and explain any amendments to information provided at registration or in the protocol. | Manuscript page 4 |
| Support | 25 | Describe sources of financial or non-financial support for the review, and the role of the funders or sponsors in the review. | Manuscript page 24 |
| Competing interests | 26 | Declare any competing interests of review authors. | Manuscript page 24 |
| Availability of data, code and other materials | 27 | Report which of the following are publicly available and where they can be found: template data collection forms; data extracted from included studies; data used for all analyses; analytic code; any other materials used in the review. | Manuscript page 24 |

| **Supplementary Material 2: Deviations from the study pre-registered protocol** | |
| --- | --- |
| **Protocol method** | **Deviation from protocol method, with justification** |
| We planned to use the following criteria to evaluate indirectness of evidence for GRADE.  ***Intervention*:**  ≥50% of studies did not report all components of the FITT principle (frequency, intensity, time, type) | The assessment of indirectness of evidence for the GRADE criteria was updated to include population, comparator, and outcomes, in addition to intervention. This was to ensure the evidence assessed was relevant to the population, allowed for a true evaluation of the effects of the intervention, and that outcomes were assessed using appropriate measures. The below criteria were added and used to assess indirectness of evidence for each outcome.  ***Population:***  ≥50% of the included studies investigated the same type of cancer.  ***Comparator:***  The control group received an intervention (exercise, nutritional or mind-body).  ***Outcomes:***  Self-Reported Outcomes: ≥50% of included studies did not use a validated single-item or multi-item questionnaire to measure self-reported outcomes such as the FACT and EORTC.  Physical Function outcomes: ≥50% of the included studies did not use a well-established measure of physical function, such as the Short Physical Performance Battery, Sit-to-Stand Test, Timed Up and Go, gait speed, 400m walk text or the 6-Minute Walk Test.  Muscular Strength outcomes: ≥50% of outcomes included in the meta-analysis involved an assessment that does not mimic the muscle actions involved in the interventions (e.g. interventions involve dynamic muscle contractions, but assessment involves an isometric contraction) |

| **Supplementary Material 3. PRISMA-S Checklist** | | | |
| --- | --- | --- | --- |
| **Section/topic** | **#** | **Checklist item** | **Location(s) Reported** |
| **INFORMATION SOURCES AND METHODS** | | | |
| Database name | 1 | Name each individual database searched, stating the platform for each. | Manuscript page 4 |
| Multi-database searching | 2 | If databases were searched simultaneously on a single platform, state the name of the platform, listing all of the databases searched. | NA |
| Study registries | 3 | List any study registries searched. | NA |
| Online resources and browsing | 4 | Describe any online or print source purposefully searched or browsed (e.g., tables of contents, print conference proceedings, web sites), and how this was done. | NA |
| Citation searching | 5 | Indicate whether cited references or citing references were examined, and describe any methods used for locating cited/citing references (e.g., browsing reference lists, using a citation index, setting up email alerts for references citing included studies). | Manuscript page 6 |
| Contacts | 6 | Indicate whether additional studies or data were sought by contacting authors, experts, manufacturers, or others. | NA |
| Other methods | 7 | Describe any additional information sources or search methods used. | NA |
| **SEARCH STRATEGIES** | | | |
| Full search strategies | 8 | Include the search strategies for each database and information source, copied and pasted exactly as run. | Supplementary material 3 |
| Limits and restrictions | 9 | Specify that no limits were used, or describe any limits or restrictions applied to a search (e.g., date or time period, language, study design) and provide justification for their use. | Supplementary material 3 |
| Search filters | 10 | Indicate whether published search filters were used (as originally designed or modified), and if so, cite the filter(s) used. | Supplementary material 3 |
| Prior work | 11 | Indicate when search strategies from other literature reviews were adapted or reused for a substantive part or all of the search, citing the previous review(s). | NA |
| Updates | 12 | Report the methods used to update the search(es) (e.g., rerunning searches, email alerts). | NA |
| Dates of searches | 13 | For each search strategy, provide the date when the last search occurred. | Supplementary material 3 |
| **PEER REVIEW** | | | |
| Peer review | 14 | Describe any search peer review process. | NA |
| **MANAGING RECORDS** | | | |
| Total Records | 15 | Document the total number of records identified from each database and other information sources. | Manuscript page 9  (OSF; <https://osf.io/yg4jd/>)  Search results |
| Deduplication | 16 | Describe the processes and any software used to deduplicate records from multiple database searches and other information sources. | Manuscript page 6 |
|  |  |  |  |
|  |  |  |  |
|  |  |  |  |
|  |  |  |  |

| **Supplementary Material 4. Search strategy**  Search terms used in PubMed, Cochrane Central Register of Controlled Trials (CENTRAL), CINAHL, SCOPUS and Web of Science. | |
| --- | --- |
|  | **PubMed (NCBI)** |
| 1 (All fields) | "Oncology" OR “Cancer” OR “Neoplasm” |
|  | AND |
| 2 (All fields) | "Resistance training" OR "Resistance exercise" OR "Strength training" OR "Strength exercise" OR "Weightlifting" OR "Weight training" |
|  | AND |
| 3 (All fields) | “Chemotherapy” OR “Chemotherapeutic” |
|  | AND |
| 4 (All fields) | “Quality of Life” OR “Fatigue” OR “Physical Function” OR “Strength” OR “Power” |
| Filters | English language |
| Limits | None |
| Search date | 18th September 2023 |
|  | **Cochrane CENTRAL (Wiley)** |
| 1 (All text) | "Oncology" OR “Cancer” OR “Neoplasm” |
|  | AND |
| 2 (All text) | "Resistance training" OR "Resistance exercise" OR "Strength training" OR "Strength exercise" OR "Weightlifting" OR "Weight training" |
|  | AND |
| 3 (All text) | “Chemotherapy” OR “Chemotherapeutic” |
|  | AND |
| 4 (All text) | “Quality of Life” OR “Fatigue” OR “Physical Function” OR “Strength” OR “Power” |
| Filter | English language |
| Limits | None |
| Search date | 18th September 2023 |
|  | **CINAHL (via EBSCOhost)** |
| 1 (All fields) | "Oncology" OR “Cancer” OR “Neoplasm” |
|  | AND |
| 2 (All fields) | "Resistance training" OR "Resistance exercise" OR "Strength training" OR "Strength exercise" OR "Weightlifting" OR "Weight training" |
|  | AND |
| 3 (All fields) | “Chemotherapy” OR “Chemotherapeutic” |
|  | AND |
| 4 (All fields) | “Quality of Life” OR “Fatigue” OR “Physical Function” OR “Strength” OR “Power” |
| Filters | English |
| Limits | None |
| Search date | 18th September 2023 |
|  | **SCOPUS (via Elsevier)** |
| 1 | "Oncology" OR “Cancer” OR “Neoplasm” |
|  | AND |
| 2 | "Resistance training" OR "Resistance exercise" OR "Strength training" OR "Strength exercise" OR "Weightlifting" OR "Weight training" |
|  | AND |
| 3 | “Chemotherapy” OR “Chemotherapeutic” |
|  | AND |
| 4 | “Quality of Life” OR “Fatigue” OR “Physical Function” OR “Strength” OR “Power” |
| Filters | Article, abstract, keywords and English |
| Limits | None |
| Search date | 18th September 2023 |
|  | **Web of Science (via Clarivate)** |
| 1 (Topic) | "Oncology" OR “Cancer” OR “Neoplasm” |
|  | AND |
| 2 (Topic) | "Resistance training" OR "Resistance exercise" OR "Strength training" OR "Strength exercise" OR "Weightlifting" OR "Weight training" |
|  | AND |
| 3 (Topic) | “Chemotherapy” OR “Chemotherapeutic” |
|  | AND |
| 4 (Topic) | “Quality of Life” OR “Fatigue” OR “Physical Function” OR “Strength” OR “Power” |
| Filters | English language |
| Limits | None |
| Search date | 18th September 2023 |

**Supplementary Material 5: Near miss studies with justification**

| **Reference** | **Title** | **Reason** |
| --- | --- | --- |
| Cešeiko et al. 2019 [1] | The impact of maximal strength training on quality of life among women with breast | Chemotherapy and/or other treatment modality |
| Cešeiko et al. 2020 [2] | Heavy resistance training in breast cancer patients undergoing adjuvant therapy | Chemotherapy and/or other treatment modality |
| Cheng et al. 2021[3] | Effect of Tai Chi and Resistance Training on Cancer-Related Fatigue and Quality of Life in Middle-Aged and Elderly Cancer Patients | Chemotherapy and/or other treatment modality |
| Eisenhut et al. 2022 [4] | Effects of two types of exercise training on psychological well-being, sleep and physical fitness in patients with high-grade glioma | Chemotherapy and/or other treatment modality |
| Hu & Zhao. 2020 [5] | Effects of resistance exercise on complications, cancer-related fatigue and quality of life in nasopharyngeal carcinoma patients undergoing chemoradiotherapy: a randomised controlled trial | Chemotherapy and/or other treatment modality |
| Kilbreath et al. 2012 [6] | Upper limb progressive resistance training and stretching exercises following surgery for early breast cancer: a randomized controlled trial | Chemotherapy and/or other treatment modality |
| Loh et al. 2020 [7] | Effects of a Home-based Exercise Program on Anxiety and Mood Disturbances in Older Adults with Cancer Receiving Chemotherapy | Combined aerobic and resistance training |
| Schmidt et al. 2015 [8] | Effects of resistance exercise on fatigue and quality of life in breast cancer patients undergoing adjuvant chemotherapy: A randomized controlled trial | Active control group |
| Wehrle et al. 2019 [9] | Endurance and resistance training in patients with acute leukemia undergoing induction chemotherapy | Active control group |

| **Supplementary Material 6. Summary of intervention characteristics** | | | | | | | |
| --- | --- | --- | --- | --- | --- | --- | --- |
| **Author(s) (year)** | **Type and setting** | **Intervention Duration (weeks)** | **Frequency (x/week)** | **Session Duration (Mins)** | **Intensity** | **Progression** | **Adherence (%)** |
| Christensen et al. (2014a) | **Type:** Machine based resistance training  **Supervised:** Yes  **Setting:** Hospital  **Exercise selection:** Leg press, knee extension, chest press and lateral pulldown. | 9 weeks | 3 times per week | Not reported | **Initial five sessions:**  3 sets of 15 repetitions  at 15 RM load  **From session six onward:**  4 sets of 10 repetitions at 10–12 RM load. | Resistance was increased when participants completed more than 12 repetitions. | Ten patients (66%) in the INT group performed ≥14 RT sessions with mean adherence of 22.6 sessions (84%). |
| Christensen et al. (2014b) | **Type:** Machine based resistance training  **Supervised:** Yes  **Setting:** Hospital  **Exercise selection:** Leg press, knee extension, chest press and lateral pulldown. | 9 weeks | 3 times per week | Not reported | **Initial five sessions:**  Three sets of 15 repetitions  at 15 RM load  **From session six onward:**  Four sets of 10 repetitions at 10–12 RM load. | Resistance was increased when participants completed more than 12 repetitions. | Mean adherence rate to RT was 18.9 sessions (70%) |
| Courneya et al. (2007) | **Type:** Machine based resistance training  **Supervised:** Yes  **Setting:** Not reported  **Exercise selection:** Leg extensions, leg curls, leg press, calf raises, chest press, seated rows, triceps extensions, biceps curls, and modified curl-ups. | 17 weeks (95% CI, 9 to 24 weeks) | 3 times per week | Not reported | Two sets of 8 to 12 repetitions of nine different exercises at 60% to 70% of their estimated one repetition maximum. | Resistance was increased by 10% when participants completed more than 12 repetitions. | RT 68.2% (2,810 of 4,079 sessions) |
| Müller et al. (2021) | **Type:** Machine based resistance training  **Supervised:** 2x supervised (exercise oncology training facility) and 1x unsupervised (home-based).  **Setting:** Exercise oncology training facility and home  **Exercise selection:**  **Supervised machine-based resistance training exercises**  Leg press, knee extension and flexion, rowing, lateral pull down, shoulder internal and external rotation, butterfly, butterfly reverse.  **Home-based core stability exercises**  15 different core stability exercises in various positions: supine position, prone position, quadruped position, plank position. | 20 weeks (20.3 ± 5.5) | 3 times per week  (2x supervised and 1x home-based session) | Total training duration per week [min]  **Supervised**  68.6 ± 17.8  **Home-based**  17 ± 6.1 | **Supervised**  Start at 70-80% 1RM  **Home-based**  RPE 14-16 | Resistance was increased when participants achieved 3 set of 12 repetitions in three consecutive training sessions. | Mean attendance: 49% in the RT group. |
| Schmidt et al. (2015) | **Type:** Machine based resistance training  **Supervised:** Yes  **Setting:** Not reported  **Exercise selection:** Squat, chest press, leg curl, rowing, leg extension, upper arm curl, upper arm extensors, shoulder press, abdominal bench and latissimus pull down. | 12 weeks | 2 times per week | 60 minutes | 1 set of 20 repetitions at 50% of hypothetic one-repetition maximum | Increase in exercise intensity were based on the Borg scale | Not reported |
| Schwartz & Winter-stone, (2009) | **Type:** Resistance band or weight equipment  **Supervised:** No  **Setting:** Home-based  **Exercise selection**: 3 to 4 upper body and lower body exercises. | 26 weeks | 4 times per week | 20 minutes | Based on each subject’s 1-repetition maximum (1RM)  3 sets of 12 reps or 2 sets of 18 to 20 reps (varied over the course of the study). | Resistance was increased upon achievement of prescribed rep and set targets.  Exercises, as well as the number of sets and repetitions, were changed periodically (every 3 to 6 months). | 74% |
| Schwartz et al, (2007) | **Type:** Resistance bands  **Supervised:** No  **Setting:** Home-based  **Exercise selection:** Two different sets of eight exercises (four upper body and four lower body) that targeted the major muscle groups used in everyday activities. | 26 weeks | 4 times per week | Not reported | Subjects were given two different sets of exercises and were asked to complete two sets of 8–10 repetitions and alternate the exercise sets within each week. | When participants were able to complete 2 sets of 10 repetitions, they were instructed to increase the resistance on the Thera-Band by modifying the starting grip position or using a band with greater resistance. | Not reported |
| Wiskemann et al. (2019)  (Supervised) | **Type:** Machine-based resistance training  **Supervised:** Yes  **Setting:** Exercise facility university campus  **Exercise selection:** Leg press, leg extension, leg curl, seated row, latissimus pull-down, back extension, butterfly reverse, and crunch. | 26 weeks | 2 times per week | 60 minutes | **Weeks 1-4 (Adaptation Period):**  Patients performed the first 5 exercises.  1 to 2 sets with 20 repetitions per exercise.  Intensity: 50%–60% of 1-RM.  **From Week 5 Onwards:**  Number of exercises increased to all 8 exercises per session.  3 sets of 8 to 12 repetitions per exercise.  Intensity: 60%–80% of 1RM. | Resistance increased by at least 5% after achieving 3 sets of 12 repetitions in three consecutive sessions. | 64.1% |
| Wiskemann et al. (2019)  (Home-based) | **Type:** Resistance bands and dumbbells  **Supervised:** No  **Setting:** Home-based  **Exercise selection**: Resistance exercises corresponded to supervised exercises of Wiskemann et al. (2019). | 26 weeks | 2 times per week | 60 minutes | **Weeks 1-4 (Adaptation Period):**  Patients performed 5 exercises.  Intensities ranged from low to moderate.  1–2 sets with 20 repetitions.  **From Week 5 Onwards:**  Number of exercises increased to 8 exercises per session.  3 sets of 8 to 12 repetitions. | Intensity was adapted using the RPE Scale (BORG Scale) with target scores of 14 to 16. If necessary, adaptations regarding exercise difficulty level were provided. | 78.4% |

CI = Confidence Interval, RT = Resistance Training, INT = Intervention, RPE = Rating of Perceived Exertion, RM = Repetition Maximum

**Supplementary Material 7. Funnel Plots**


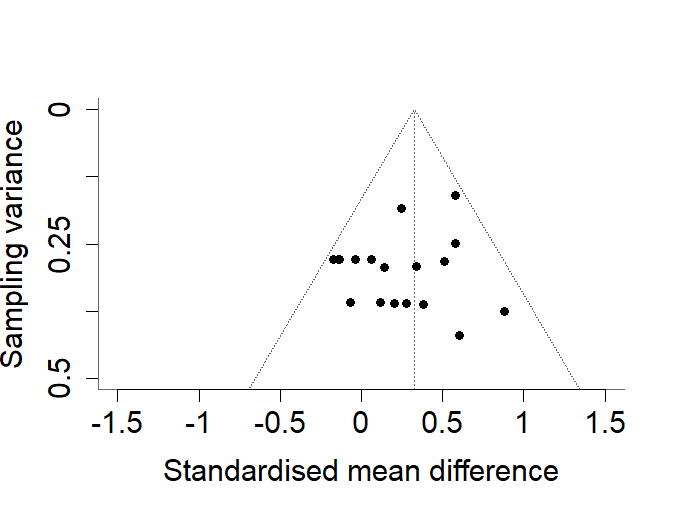

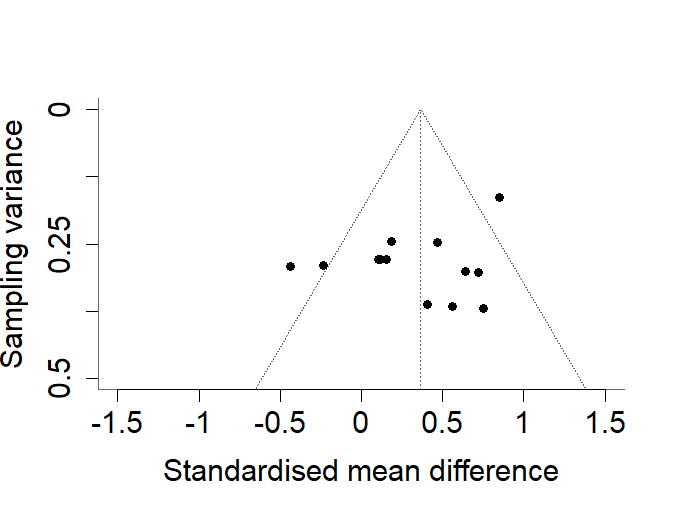


(B)

(A)

Funnel plot of the standardized mean differences from individual studies against the corresponding sampling variances for upper-body strength (A) and lower-body strength (B)

**References**

1. Cešeiko R, Eglītis J, Srebnijs A, et al (2019) The impact of maximal strength training on quality of life among women with breast cancer undergoing treatment. Exp Oncol 41:166–172. https://doi.org/10.32471/exp-oncology.2312-8852.vol-41-no-2.13249

2. Cešeiko R, Thomsen SN, Tomsone S, et al (2020) Heavy Resistance Training in Breast Cancer Patients Undergoing Adjuvant Therapy. Med Sci Sports Exerc 52:1239–1247. https://doi.org/10.1249/MSS.0000000000002260

3. Cheng D, Wang X, Hu J, et al (2021) Effect of Tai Chi and Resistance Training on Cancer-Related Fatigue and Quality of Life in Middle-Aged and Elderly Cancer Patients. Chin J Integr Med 27:265–272. https://doi.org/10.1007/s11655-021-3278-9

4. Eisenhut L, Sadeghi-Bahmani D, Gerber M, et al (2022) Effects of two types of exercise training on psychological well-being, sleep and physical fitness in patients with high-grade glioma (WHO III and IV). J Psychiatr Res 151:354–364. https://doi.org/10.1016/j.jpsychires.2022.03.058

5. Hu Q, Zhao D (2021) Effects of resistance exercise on complications, cancer-related fatigue and quality of life in nasopharyngeal carcinoma patients undergoing chemoradiotherapy: A randomised controlled trial. Eur J Cancer Care (Engl) 30:e13355. https://doi.org/10.1111/ecc.13355

6. Kilbreath SL, Refshauge KM, Beith JM, et al (2012) Upper limb progressive resistance training and stretching exercises following surgery for early breast cancer: a randomized controlled trial. Breast Cancer Res Treat 133:667–676. https://doi.org/10.1007/s10549-012-1964-1

7. Loh KP, Kleckner IR, Lin P-J, et al (2019) Effects of a Home-based Exercise Program on Anxiety and Mood Disturbances in Older Adults with Cancer Receiving Chemotherapy. J Am Geriatr Soc 67:1005–1011. https://doi.org/10.1111/jgs.15951

8. Schmidt ME, Wiskemann J, Armbrust P, et al (2015) Effects of resistance exercise on fatigue and quality of life in breast cancer patients undergoing adjuvant chemotherapy: A randomized controlled trial. Int J Cancer 137:471–480. https://doi.org/10.1002/ijc.29383

9. Wehrle A, Kneis S, Dickhuth H-H, et al (2019) Endurance and resistance training in patients with acute leukemia undergoing induction chemotherapy—a randomized pilot study. Support Care Cancer 27:1071–1079. https://doi.org/10.1007/s00520-018-4396-6
